# Endemic chronic wasting disease causes mule deer population decline in Wyoming

**DOI:** 10.1371/journal.pone.0186512

**Published:** 2017-10-19

**Authors:** Melia T. DeVivo, David R. Edmunds, Matthew J. Kauffman, Brant A. Schumaker, Justin Binfet, Terry J. Kreeger, Bryan J. Richards, Hermann M. Schätzl, Todd E. Cornish

**Affiliations:** 1 Department of Veterinary Sciences, University of Wyoming, Laramie, Wyoming, United States of America; 2 Natural Resource Ecology Laboratory, Colorado State University/US Geological Survey, Fort Collins, Colorado, United States of America; 3 U.S. Geological Survey, Wyoming Cooperative Fish and Wildlife Research Unit, Department of Zoology and Physiology, University of Wyoming, Laramie, Wyoming, United States of America; 4 Wyoming Game and Fish Department, Casper, Wyoming, United States of America; 5 Wyoming Game and Fish Department, Wheatland, Wyoming, United States of America; 6 U.S. Geological Survey, National Wildlife Health Center, Madison, Wisconsin, United States of America; 7 Department of Comparative Biology & Experimental Medicine, University of Calgary, Calgary, Alberta, Canada; Colorado State University College of Veterinary Medicine and Biomedical Sciences, UNITED STATES

## Abstract

Chronic wasting disease (CWD) is a fatal transmissible spongiform encephalopathy affecting white-tailed deer (*Odocoileus virginianus*), mule deer (*Odocoileus hemionus*), Rocky Mountain elk (*Cervus elaphus nelsoni*), and moose (*Alces alces shirasi)* in North America. In southeastern Wyoming average annual CWD prevalence in mule deer exceeds 20% and appears to contribute to regional population declines. We determined the effect of CWD on mule deer demography using age-specific, female-only, CWD transition matrix models to estimate the population growth rate (*λ*). Mule deer were captured from 2010–2014 in southern Converse County Wyoming, USA. Captured adult (≥ 1.5 years old) deer were tested ante-mortem for CWD using tonsil biopsies and monitored using radio telemetry. Mean annual survival rates of CWD-negative and CWD-positive deer were 0.76 and 0.32, respectively. Pregnancy and fawn recruitment were not observed to be influenced by CWD. We estimated *λ* = 0.79, indicating an annual population decline of 21% under current CWD prevalence levels. A model derived from the demography of only CWD-negative individuals yielded; *λ* = 1.00, indicating a stable population if CWD were absent. These findings support CWD as a significant contributor to mule deer population decline. Chronic wasting disease is difficult or impossible to eradicate with current tools, given significant environmental contamination, and at present our best recommendation for control of this disease is to minimize spread to new areas and naïve cervid populations.

## Introduction

Chronic wasting disease (CWD) is a fatal transmissible spongiform encephalopathy affecting white-tailed deer (*Odocoileus virginianus*), mule deer (*Odocoileus hemionus*), Rocky Mountain elk (*Cervus elaphus nelsoni*), and moose (*Alces alces shirasi)* in North America [1–5]. All transmissible spongiform encephalopathies are caused by unconventional infectious agents composed of the proteinase-resistant pathologic isoform (PrP^res^) of the normal cellular prion protein (PrP^C^) [6–8]. Chronic wasting disease naturally occurs in free-ranging cervid populations in 21 U.S. states and two Canadian provinces [9], but limited information exists regarding the population-level impacts of CWD in the wild. In captivity, annual CWD incidence may exceed 50% in mule deer and white-tailed deer [10] and epidemics often end in the depopulation of deer at research facilities. Declines in free-ranging mule deer in Table Mesa, Colorado, elk in Rocky Mountain National Park in Colorado, and white-tailed deer in southeastern Wyoming were attributed to CWD prevalence greater than 13% [11–13]. From 2001–2009, the Wyoming Game and Fish Department (WGFD) recorded an average CWD prevalence of 31% from hunter harvested mule deer in southern Converse County, Wyoming [14]. Concurrently, WGFD estimated a >50% reduction in the South Converse Mule Deer Herd [SCMDH; 14]. High annual CWD prevalence and declining population trends in this mule deer herd warranted investigation of the influence of CWD-associated declines in vital rates (i.e. survival, pregnancy, and fawn recruitment) and population growth rate (*λ*).

We hypothesized that CWD negatively impacted adult survival, pregnancy, and recruitment of fawns. The effect of CWD on population growth was measured by estimating *λ* for the CWD test-postive and test-negative portions of the population. Prior research revealed mule deer possessing at least one phenylalanine (F) at codon 225 of the prion protein gene (*Prnp*) were less susceptible to CWD infection compared to homozygous serine (S) genotyped deer [15]. Therefore, we evaluated the influence of *Prnp* on CWD incidence and compared *λ* estimates of the phenylalanine genotype (225SF and 225FF deer grouped and hereafter referred to as 225*F deer) and homozygous serine genotype (225SS) segments of the population [15]. Other studies suggested CWD-positive deer are more likely to be killed by mountain lions (*Puma concolor*) [16] and that mountain lions may selectively prey on prion-infected deer [17]. Mule deer also may be more vulnerable to vehicle collisions, especially during the later stages of infection [18]. Sympatric CWD-positive white-tailed deer were more likely to be harvested by hunters [13]. Thus, we evaluated if CWD-positive deer were more susceptible to specific causes of mortality.

## Material and methods

### Study area and population

We studied mule deer from the SCMDH that wintered primarily within the LaPrele Valley that surrounds the LaPrele Reservoir in southern Converse County, Wyoming from 2010–2014. The aggregate home range of all marked mule deer occupied an area ~2,576 km^2^. Deer wintered at elevations of ~1,500 m and a portion of the population migrated to summer ranges at ~2,700 m. Our study area was predominantly comprised of private native rangelands with some cultivated meadows along with some small tracts of public land. Some mule deer seasonally migrated to higher elevations where larger tracts of national forest occurs. True mountain mahogany (*Cercocarpus montanus*), antelope bitterbrush (*Purshia tridentata*), and big sagebrush (*Artemisia tridentata*) dominated the foothills while sagebrush and irrigated hayfields dominated the lowland areas. In 2010, the WGFD estimated the SCMDH at ~6,100 deer and by the conclusion of the study in 2014, the herd was estimated at ~5,100 deer based on post-harvest population estimates and different modeling techniques for 2010 (POP-II, Fossil Creek Software) and 2011–2014 (spreadsheet model) [19]. The hunting of does and fawns was largely eliminated in 2009 in response to poor population performance [14,19]. Throughout the course of this study, a seven-day general antlered mule deer season occurred in this population with approximately 300 males harvested each year within the herd unit [19]. Annual CWD prevalence of sympatric male and female white-tailed deer and elk harvested during the study averaged 13.32% (n = 42) and 5.92% (n = 529), respectively.

### Captures and field data collection

We aerial net-gunned adult mule deer during winter (February/March) [20], focusing primarily on females and capturing at least 40 females each year. Males were captured from 2011–2014 to evaluate sex-associated CWD prevalence and survival. Captured deer were chemically immobilized with an intramuscular (IM) injection of either 0.03 mg/kg of carfentanil and 0.7 mg/kg of xylazine or 0.5 mg/kg of butorphanol, 0.35 mg/kg of azaperone, and 0.22 mg/kg of medetomidine (BAM; [21,22]). We collected blood by jugular venipuncture and used it for *Prnp* determination using restriction fragment length polymorphism with confirmation by sequencing of PCR fragments of random samples [15]. Serum separated from blood samples was used for pregnancy analysis by pregnancy-specific protein B (PSPB) concentration (BioTracking LLC, Moscow, Idaho, USA). Approximate age at capture was determined using tooth eruption and wear [23]. Incisors from recovered carcasses were aged using cementum annuli analysis [24]. We assessed body condition by assigning a subjective score based on palpation of fat and muscle. Tonsil biopsies were performed to test deer for CWD by immunohistochemistry (IHC) and surgical equipment was cleaned using methods previously published to prevent iatrogenic transmission [25]. Deer were administered subcutaneous procaine/benzathine penicillin G combination (25,000 units/kg based on benzathine fraction, Bimeda, Le Sueur, Minnesota, USA) and 1.5 mg/kg of Banamine IM (Intervet Inc., Merck Animal Health, Summit, New Jersey, USA). Deer were given naltrexone (100 mg/mg of carfentanil) and tolazoline (2mg/kg) or naltrexone (50–100 mg), tolazoline (200–300 mg), and atipamezole (15–25 mg) to reverse anesthetic effects of carfentanil/xylazine or BAM, respectively [21,22]. Deer were fitted with either a store-onboard global positioning system (GPS) radio-collar (Lotek Wireless Inc., Newmarket, Ontario, Canada) or a very-high-frequency (VHF) radio-collar (Advanced Telemetry Systems, Inc., Isanti, Minnesota, USA) equipped with mortality signal that was activated after 4 hours of inactivity. Deer were tagged with a large cattle ear-tag with an identification number and contact information if harvested for postmortem CWD testing and a metal WGFD identification ear-tag. Surviving deer were recaptured annually and processed as described with the exception of known CWD-positive deer, which no longer required biopsy. The study was completed in 2014 with the removal of radio-collars and final release of all surviving deer. All procedures involving deer were performed under the approval of the University of Wyoming Institutional Animal Care and Use Committee (No. A-3216-01) and the Wyoming Game and Fish Department (Permit No. 33–751).

Radio-collared mule deer were monitored at least twice weekly and mortalities were recovered to determine cause of death. Mortalities were investigated immediately after detection to recover carcasses prior to scavenging and autolysis. Necropsies were performed either in the field or at the Wyoming State Veterinary Laboratory. Postmortem CWD tests were performed when feasible. Retropharyngeal lymph nodes (RLNs) were collected and one was tested for PrP^res^ using the enzyme-linked immunosorbent assay (ELISA; [26]). Tonsil, obex region of the medulla oblongata, and the other RLN were tested for PrP^res^ by IHC [27]. We determined cause of death as clinical CWD if a deer tested positive for CWD postmortem and presented with no other signs of disease and trauma. While CWD is a fatal disease, not all CWD-positive deer died due to clinical disease. The proximate cause of death was recorded for each mortality case regardless of CWD status at the time of necropsy.

Fawn recruitment (number of fawns per marked doe) was documented during November ground surveys from 2011–2013. Females that were pregnant during captures were located via telemetry. If fawns were not seen with marked females during the initial observation, females were displaced to observe fawns fleeing the area. If no fawns were observed during the first attempt, subsequent attempts were made until the end of November.

### Kaplan-Meier survival and incidence analysis

Annual survival and incidence were estimated using Kaplan-Meier known-fates estimation [28,29]. Survival was estimated from previous capture event (*t*-1) to current capture event (*t*) and all deer entered the study analysis at *t* = 0 regardless of initial capture year. Survival and incidence were determined based on biological year (June 1^st^–May 31^st^), which formed the basis of stage-structured Lefkovitch matrix models [30]. Daily survival and weekly CWD incidence were calculated using the *survival* package v.2.37–7 [31] and *survfit* function in statistical program R v.3.1.0 [32]. Mortality dates were determined as the first mortality event recorded by the GPS radio-collar following initial capture. Mortality date for deer tagged with VHF radio-collars was determined either by the condition of the carcass or as the midpoint date between a live observation and a dead observation. Deer were right censored if they were lost to follow-up due to relocation failure, died from unnatural causes such as poaching or capture-related mortality, or survived to the end of the study. Several deer started the study as CWD-negative and subsequently tested positive, thus their survival time was split into two datasets, in which they were right censored as a CWD-negative deer at their CWD-positive test date. Survival estimates of CWD-positive deer used in our analyses were potentially biased low because deer were considered CWD-positive the day they tested positive and we included deer that were initially captured as test-positive animals. Post hoc survival analysis revealed that our survival estimate that included all CWD-positive deer fell within the 95% CI (0.28, 0.69) of 26 deer that experienced a CWD incident event during the study (we left censored 17 deer without known fates because they were test-positive post-mortem or test-positive during the last capture when monitoring ceased). Survival was determined separately based on sex, CWD status, age-class, and *Prnp* genotype. Deer were left censored when calculating incidence if they were initially CWD-positive. An incident event occurred when a CWD-negative deer first tested CWD-positive and right censored if lost to follow-up, CWD status was not determined on subsequent captures, or study ended with a final CWD-negative test. Incidence was calculated separately based on sex, age-class, and *Prnp* genotype. Log-rank tests [29,33] were performed in R using the function *survdiff* to compare all Kaplan-Meier curves [31].

### Extended Cox proportional hazards model analysis

We examined the effects of sex, age at capture, CWD status, and *Prnp* genotype on weekly survival probability. An extended Cox proportional hazards model was used to determine which variables had the most influence on annual survival of deer [29,34]. The analyses were performed using the *coxph* function in R [31]. Time-dependent variables were created for CWD status and age as both changed through time for individual deer during the study [29]. Proportional hazards assumption was tested using the *cox*.*zph* function, which evaluates correlation between the Schoenfeld residuals and survival time [31]. Covariates failed proportionality when their p-value ≤ 0.05 [29]. Stepwise forward and backward selection of models were performed using the function *stepAIC* in the package *MASS* v.7.3–31 [35]. Models were ranked based on Akaike’s Information Criteria (AIC) values [36]. Model AIC values within 2 AICs of the best model (Δ AIC) were considered good predictors of survival and individual covariate p-values were evaluated for final model selection [36].

### Pregnancy and recruitment mixed model analysis

We used generalized linear mixed models to determine the effects of age, CWD status, winter body condition, *Prnp* genotype, and observation year on annual proportion pregnant deer and fawn recruitment. A repeated measures analysis was performed and data grouped by unique deer identification was modeled using the *glmer* function in program *lme4* v.1.1–7 [37]. Pregnancy and recruitment indices were calculated separately based on CWD status and observation year.

### Population growth rate estimation

**Fig 1:**
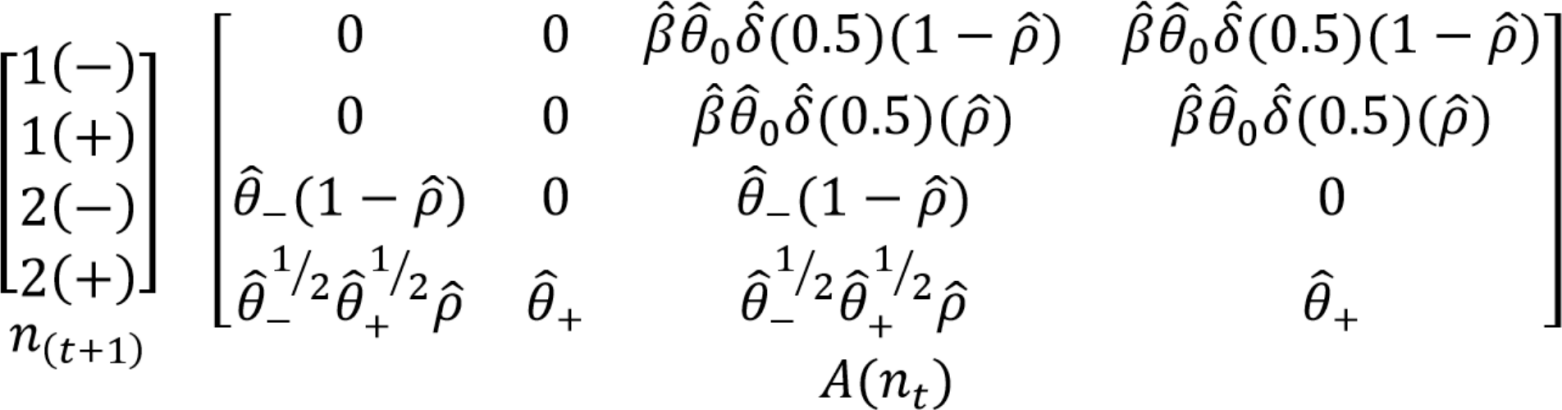
Lefkovitch matrix model A representing transition of a female-only, pre-breeding, chronic wasting disease-structured 4 x 4 matrix of a mule deer population in southern Converse County, WY using demographic and disease rates observed from 2010–2014. n_t_ represents the number of deer in each age class by CWD status (-; PrP^CWD^ not detected and +; PrP^CWD^ detected). ${\hat{\theta }}_{\left(-,+\right)}$ represents estimated survival of CWD-negative or CWD-positive deer and ${\hat{\theta }}_{\left(0\right)}$ is the estimated fawn survival from mid-December to mid-June. $\hat{\rho }$ represents CWD incidence, $\hat{\beta }$ is the estimated pregnancy rate, and $\hat{\delta }$ is the estimated recruitment rate determined in November.

An age- and CWD-structured, female-only Lefkovitch matrix model was used to estimate *λ* in MATLAB^®^ (The MathWorks, Inc., Natick, MA, USA; [38]. We used a pre-breeding census, in which deer were counted prior to the birth-pulse in June, thus the first age-class in our model was yearling. Our matrix (Fig 1) represented both CWD-negative and CWD-positive deer of age *x*_*i*_, where a deer could survive to age *x*_*i*_ + 1 at a probability of ${\hat{\theta }}_{i-}(1-{\hat{\rho }}_{i})$, where ${\hat{\theta }}_{i-}$ was the probability of a CWD-negative deer surviving and $\left(1-{\hat{\rho }}_{i}\right)$ was the transition probability of remaining CWD-negative. Deer that were CWD-negative survived and became CWD-positive at a probability of ${\hat{\theta }}_{i-}^{\frac{1}{2}}{\hat{\theta }}_{i+}^{\frac{1}{2}}({\hat{\rho }}_{i})$, which represents continuous disease transmission from time t to t+1, and CWD-positive deer survived at a probability of ${\hat{\theta }}_{i+}$.

The vital rates included in the matrix model were pregnancy ($\hat{\beta }$), fawn recruitment ($\hat{\delta }$), overwinter fawn survival (${\hat{\theta }}_{0}$), adult survival (≥ 1 year old) of CWD-negative deer (${\hat{\theta }}_{-}$), adult survival of CWD-positive deer (${\hat{\theta }}_{+}$), and CWD incidence ($\hat{\rho }$). Fawns were not captured in our study; therefore, overwinter fawn survival from mid-December to mid-June was estimated from comparable areas in Colorado, from 1997 to 2008 [39]. Due to small sample size and non-significant differences in survival and fecundity among ≥ two-year-olds, we built our matrix model to include two age-classes, yearlings and adults. We calculated the 95% confidence interval for *λ* using previously described methods of parametric bootstrapping [40,41], modified using our vital rates and matrix configuration. Overwinter fawn survival was bootstrapped using the standard deviation published for the point estimate [39]. Sensitivity and elasticity analyses were performed to evaluate how sensitive *λ* was to changes in individual vital rates using the method of Morris and Doak [41].

We initially ignored the influence of *Prnp*-genotype on CWD incidence and used the overall female-only CWD incidence during the biological year in our matrix. However, to understand the effect of CWD incidence on *λ*_,_ we varied annual incidence in the matrix from 0% to 100% and calculated the change in *λ*. Additionally, we calculated *λ* using genotype-specific incidence rates to examine estimated growth rates for the 225SS and 225*F segments of the population. The approach used to model *λ* for all scenarios assumes constant vital rates, thus density dependence was not represented in model results.

## Results

### Annual CWD prevalence and incidence

During the study, 143 mule deer were captured (118 female, 25 male) and *Prnp* genotypic frequencies were 78% 225SS deer and 22% 225*F deer. Average annual CWD prevalence was 24% (95% CI = 22%–27%). Male CWD prevalence was higher throughout the study (average = 43%) compared to female CWD prevalence (average = 18%). Seventy-seven deer tested positive for CWD during the study, of which 43 were deer that transitioned from test negative to positive. Annual CWD incidence did not differ among observation years (*χ*^*2*^ = 3.2, df = 3, p = 0.36) and did not increase suggesting iatrogenic transmission likely did not occur. Also, annual CWD incidence was not different among age-classes (*χ*^*2*^ = 8.5, df = 7, p = 0.29) and between sex for years 2011 (*χ*^*2*^ = 0.3, df = 1, p = 0.56) and 2012 (*χ*^*2*^ = 2.7, df = 1, p = 0.10). In 2013, annual CWD incidence was significantly higher in males than females (*χ*^*2*^ = 6.1, df = 1, p = 0.01). Annual CWD incidence differed among genotypes (*χ*^*2*^ = 34.5, df = 2, p < 0.01), with 225SS deer more likely to become CWD-positive compared to 225*F deer. Average annual female CWD incidence was 0.26 (SE = 0.04) and genotype-specific incidence used in our matrix models were 0.49 (SE = 0.05) for 225SS deer and 0.02 (SE = 0.06) for 225*F deer.

### Cause-specific mortality

We documented 97 mortalities of radio-collared deer. Mule deer that were CWD-positive were more susceptible to mountain lion predation (n = 20; *χ*^*2*^ = 6.36, df = 1, p = 0.01), hunter harvest (n = 4; *χ*^*2*^ = 7.98, df = 1, p < 0.01), and illegal harvest (n = 2; *χ*^*2*^ = 3.99, df = 1, p = 0.05). Mountain lion predation was the number one cause of mortality followed by clinical CWD (n = 14). Other natural causes of mortality of radio-collared mule deer included vehicle collision (n = 3), coyote predation (n = 1), fence entanglement (n = 1), drowning (n = 1), and winter kill (n = 1). Thirteen deer died due to injuries sustained during captures and we were unable to determine the cause of death in 37 cases due to severe autolysis and scavenging.

### Extended Cox proportional hazards models

Stepwise selection based on AIC values of our extended Cox proportional hazards models resulted in four competing models. The model that incorporated sex and CWD was selected as the best model for predicting survival based on individual covariate p-values (Table 1). Male mule deer were twice as likely (Hazard Ratio = 2.08, p = 0.01, 95% Confidence Interval (CI) = 1.18–3.68) to experience a mortality event compared to females and CWD-positive deer were over three times more likely (Hazard Ratio = 3.30, p < 0.0001, 95% CI = 1.98–5.49) to die during our study compared to CWD-negative deer. Genotype was included in our models initially; however, models did not converge because they lacked full representation of the *Prnp* genotypes in both CWD categories (CWD-negative and CWD-positive). Only one 225SF deer tested CWD-positive out of 29 deer, but was censored after 154 weeks and neither of the two 225FF deer captured tested CWD-positive during the study. Therefore, we removed *Prnp* genotype from model analysis and we were not able to determine the influence of genotype on CWD-positive survival probability.

**Table 1:** Extended Cox proportional hazards models with *a priori* variable selection of parameters that potentially influenced mule deer survival in southern Converse County, WY from 2010–2014.

Model	Model parameters	*K*	AIC	Δ AIC
1	Sex^a^, CWD^b^	2	557.51	0
2	Sex, CWD, Age*t	3	557.78	0.27
3	Sex, CWD, Age*t, CWD*Age*t	4	557.98	0.47
4	Sex, CWD, Age*t, Sex*CWD	4	559.03	1.52
5	CWD, Age*t	2	561.18	3.67
6	Sex, Age*t	2	577.98	20.47

*K*, number of parameters; AIC, Akaike information criterion; ΔAIC, difference with best model AIC value; CWD, chronic wasting disease; t, time

Age*t, time-dependent covariate of age

*, interaction.

^a^ Hazard Ratio = 2.08, 95% Lower Confidence Interval (LCI) = 1.18, 95% Upper Confidence Interval (UCI) = 3.68, *P* = 0.01

^b ^Hazard Ratio = 3.30, 95% LCI = 1.98, 95% UCI = 5.49, *P* < 0.01

### Annual survival estimates

**Fig 2:**
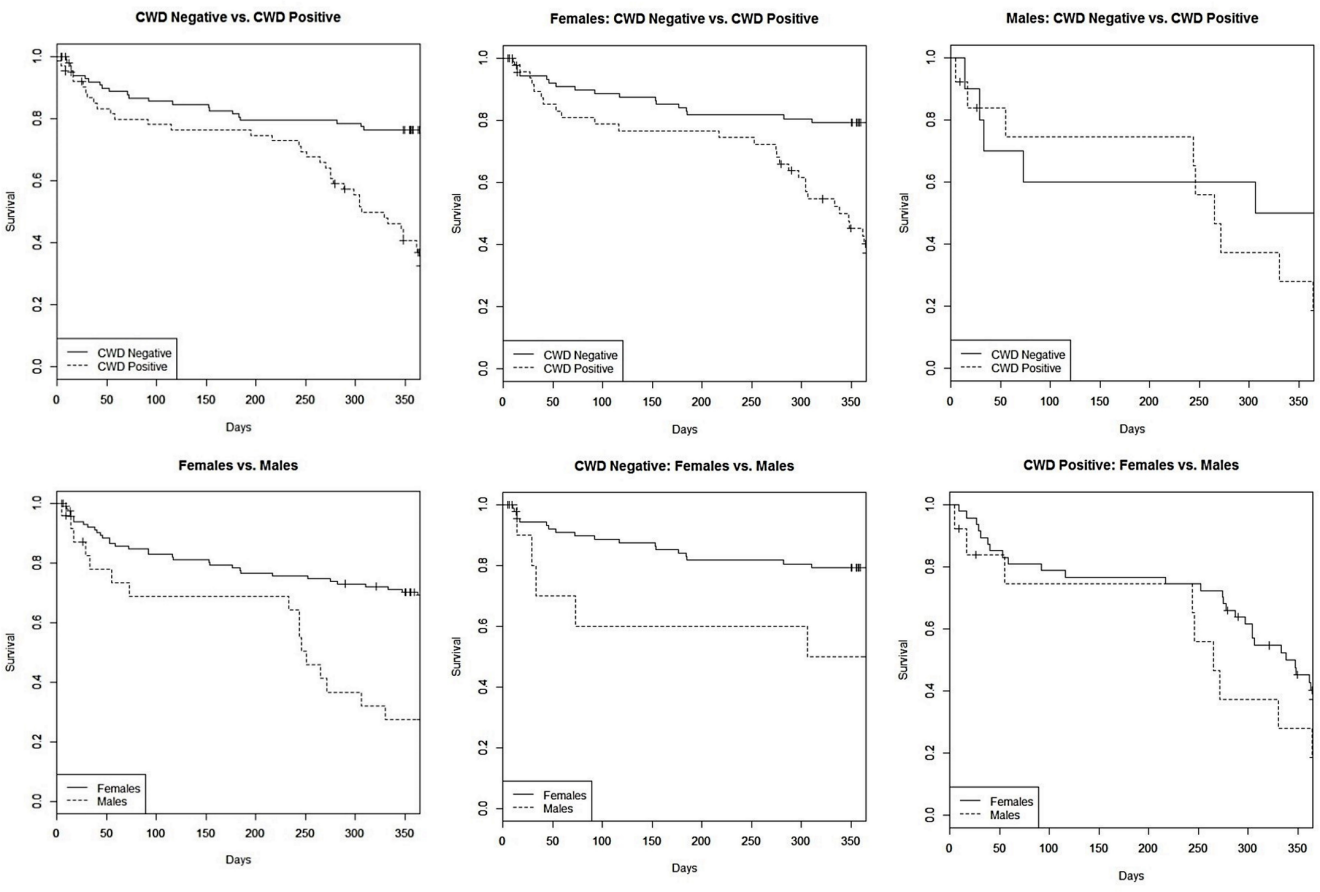
Kaplan-Meier annual survival curves of free-ranging mule deer in southern Converse County, Wyoming captured as part of a study investigating the population-level impacts of chronic wasting disease from 2010–2014.

Kaplan-Meier annual survival was significantly different between CWD-negative and CWD-positive deer (*χ*^*2*^ = 40.10, df = 2, p < 0.01), CWD-negative and CWD-positive females (*χ*^*2*^ = 38.30, df = 2, p < 0.01), and CWD-negative females and CWD-negative males (*χ*^*2*^ = 9.00, df = 2, p = 0.002; Table 2). Estimated annual survival of CWD-negative deer (0.76, SE = 0.04) was considerably higher than CWD-positive deer (0.32, SE = 0.06; Table 2). Female deer survived at a rate of 0.79 (SE = 0.04) annually compared to 0.50 (SE = 0.16) annual survival of male deer; however, this sex-associated difference was not observed for CWD-positive deer (Table 2). When comparing female and male survival curves, both declined at similar rates until a dramatic decline in male survival around 250 days that corresponded to the short hunting season (Fig 2). This accelerated rate of decline in survival curves at about 250 days was prominent when comparing CWD-negative and CWD-positive males (Fig 2). A similar pattern of accelerated decline was observed between CWD-negative and CWD-positive females starting around day 275 (Fig 2).

**Table 2:** Kaplan-Meier survival rates and log-rank test results by sex, age, and chronic wasting disease (CWD) status of mule deer in southern Converse County, WY from 2010–2014.

Category	Results	Overall	1.5	2.5	3.5	4.5	5.5+
**CWD (-) vs. (+) deer**	**Survival: CWD (-)**	0.76	0.63	0.67	0.91	0.70	1.00
	**Survival: CWD (+)**	0.32	0.00	0.15	0.60	0.28	0.51
	**χ**^**2**^	40.10	1.70	14.70	19.20	4.20	2.50
	**P-value**	0.00	0.19	0.00	0.00	0.04	0.12
**CWD (-) vs. (+) females**	**Survival: CWD (-)**	0.79	0.67	0.70	0.97	0.67	1.00
	**Survival: CWD (+)**	0.37	0.00	0.20	0.61	0.34	0.44
	**χ**^**2**^	38.30	1.10	14.50	23.20	2.20	2.90
	**P-value**	0.00	0.31	0.00	0.00	0.14	0.09
**CWD (-) vs. (+) males**	**Survival: CWD (-)**	0.50	0.50	0.33	0.50	n = 1	n = 0
	**Survival: CWD (+)**	0.19	0.00	0.00	0.50	0	n = 1
	**χ**^**2**^	1.10	0.20	0.00	0.10		
	**P-value**	0.29	0.70	0.83	0.78		
**CWD (-) females vs. males**	**Survival: females**	0.79	0.67	0.70	0.97	0.67	1.00
	**Survival: males**	0.50	0.50	0.33	0.50	n = 1	n = 0
	**χ**^**2**^	9.00	0.20	1.60	2.40		
	**P-value**	0.00	0.62	0.21	0.12		
**CWD (+) females vs. males**	**Survival: females**	0.37	0.00	0.20	0.61	0.34	0.44
	**Survival: males**	0.19	0.00	0.00	0.50	0.00	n = 1
	**χ**^**2**^	2.60	1.00	0.00	0.20	4.80	
	**P-value**	0.11	0.32	0.99	0.64	0.03	

Annual survival was not significantly different among age-classes for either CWD-negative (*χ*^*2*^ = 7.00, df = 5, p = 0.22) or CWD-positive deer (*χ*^*2*^ = 0.80, df = 4, p = 0.936). Therefore, when combining adult age-classes (≥ 2 years old) for our matrix model and survival from June 1^st^–May 31^st^ of CWD-negative females and CWD-positive females, survival was 0.85 (SE = 0.13) and 0.38 (SE = 0.34), respectively. Survival of CWD-negative females among genotypes was marginally significant (*χ*^*2*^ = 5.8, df = 2, p = 0.05) with higher survival of 225*F deer compared to 225SS deer.

### Annual pregnancy and recruitment estimates

Mean annual pregnancy of CWD-negative and CWD-positive females was 0.99 (SD = 0.11, 95% CI = 0.97–1.00) and 0.94 (SD = 0.24, 95% CI = 0.88–1.00), respectively (Table 3). Fawn recruitment from birth to November was similar between CWD-negative (average = 0.48, SD = 0.65, 95% CI = 0.33–0.63) and CWD-positive deer (average = 0.56, SD = 0.65, 95% CI = 0.30–0.82; Table 4). Age, winter body condition, CWD status, *Prnp* genotype, and observation year did not influence pregnancy and recruitment of fawns (p > 0.05).

**Table 3:** Proportion of mule deer that were pregnant at approximately 75 days bred in southern Converse County, WY.

	CWD-Negative	CWD-Positive
Year	Proportion pregnant (LCI,UCI)	Proportion pregnant (LCI, UCI)
**2010**	1.00 (1.00, 1.00)	0.88 (0.64, 1.00)
**2011**	0.97 (0.91, 1.00)	0.91 (0.73, 1.00)
**2012**	1.00 (1.00, 1.00)	0.94 (0.83, 1.00)
**2013**	1.00 (1.00, 1.00)	0.95 (0.85, 1.00)
**2014**	0.95 (0.86, 1.00)	1.00 (1.00, 1.00)
**Average**	0.99 (0.97, 1.00)	0.94 (0.88, 1.00)

LCI, 95% lower confidence interval; UCI, 95% upper confidence interval.

**Table 4:** Proportion of fawns at heel during November recruitment surveys of radio-collared female mule deer that were either CWD-test negative or positive during winter captures in southern Converse County, WY.

	CWD-Negative	CWD-Positive
Year	Fawns/Doe (LCI,UCI)	Fawns/Doe (LCI, UCI)
**2011**	0.48 (0.24, 0.72)	0.29 (0.00, 0.65)
**2012**	0.40 (0.17, 0.63)	0.56 (0.08, 1.03)
**2013**	0.56 (0.26, 0.86)	0.78 (0.34, 1.21)
**Average**	0.48 (0.33, 0.63)	0.56 (0.30, 0.82)

LCI, 95% lower confidence interval; UCI, 95% upper confidence interval.

### Population models

**Fig 3:**
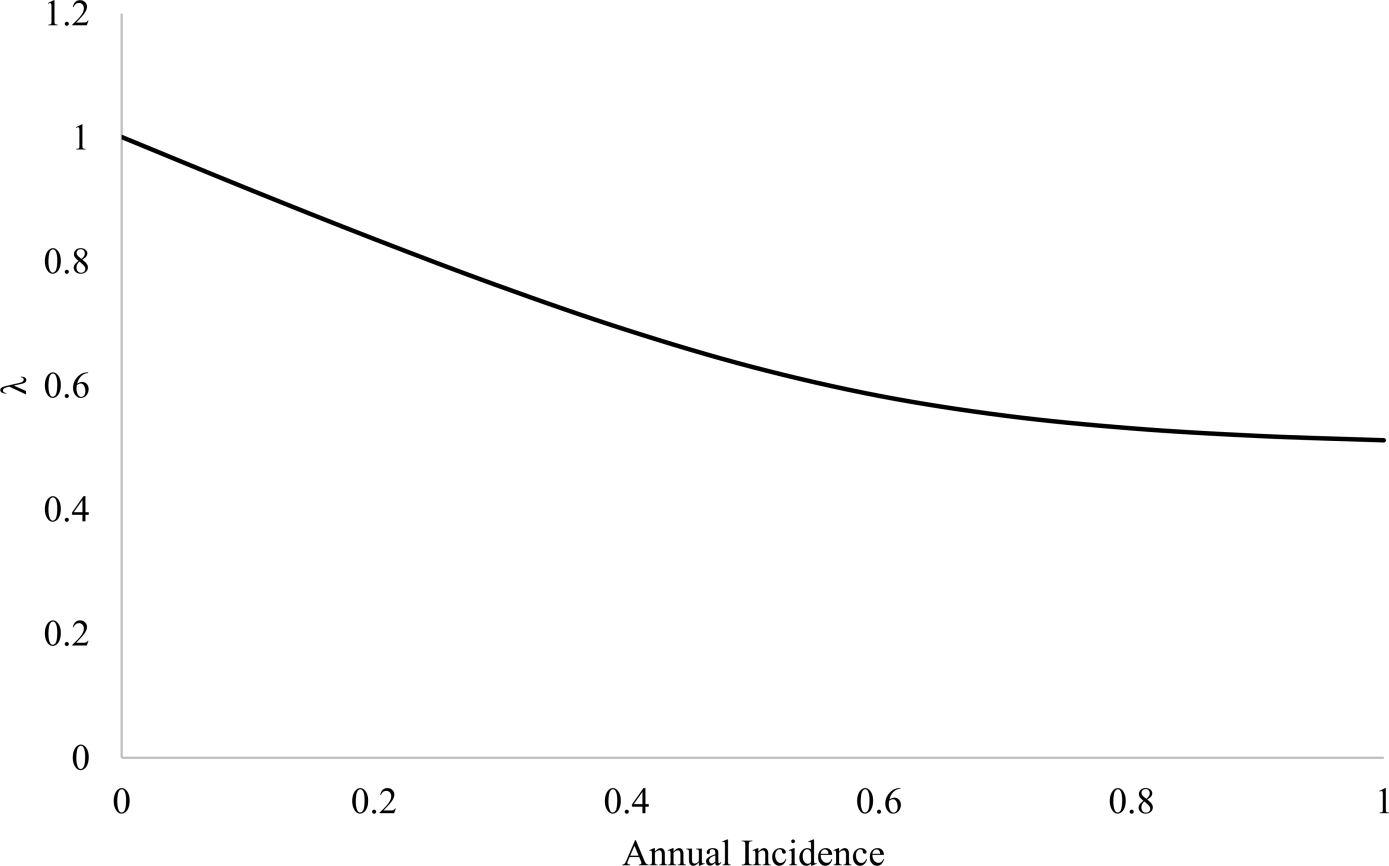
Chronic wasting disease (CWD) annual incidence and its effect on finite rate of population growth (*λ*; solid line) when all other vital rates were kept constant in our Lefkovitch matrix model of a mule deer population in southern Converse County, WY (2010–2014).

Our matrix model estimated *λ* = 0.79 (0.72, 0.87) that corresponded to a 21% annual decrease in the population with a population half-life of 4 years. The models that assumed 100% CWD prevalence and 0% CWD prevalence estimated *λ* = 0.51 and *λ* = 1.00, respectively (Fig 3). Using the estimated CWD incidence for 225SS deer, we estimated *λ* = 0.64 and for 225*F, *λ* = 0.98. The matrix model was most sensitive to changes in survival of CWD-negative deer (${\hat{\theta }}_{-}$) and CWD incidence (${\hat{P}}_{i}$; Table 5). However, when the sensitivities of vital rates were rescaled to account for proportional changes (elasticity), only changes in CWD-negative survival had largest effect on *λ* (Table 5).

**Table 5:** Sensitivities and elasticities of vital rates included in our Lefkovitch matrix model representing a mule deer population in southern Converse County, WY from 2010–2014.

Vital rate	Symbol	Estimated value	Sensitivity	Elasticity
**Pregnancy rate**	$\hat{\beta }$	0.97	0.1461	0.1753
**November recruitment**	$\hat{\delta }$	0.51	0.2779	0.1753
**Over-winter survival rate of fawns**	${\hat{\theta }}_{0}$	0.72	0.1968	0.1753
**Survival rate of CWD negative deer**	${\hat{\theta }}_{-}$	0.85	0.7126	0.7491
**Survival rate of CWD positive deer**	${\hat{\theta }}_{+}$	0.38	0.1609	0.0756
**CWD incidence rate**	${\hat{\rho }}_{i}$	0.26	-0.6939	-0.2231

## Discussion

Our findings support CWD as a population-limiting disease of mule deer with the potential to cause dramatic declines that resemble local population extinction. Other studies have found a negative association between CWD prevalence and *λ* [11,12,40,42], but none have documented *λ* estimates resulting from endemic CWD as low as those reported here. The only scenario in which population growth rate was stable (*λ* = 1) was in the absence of CWD. Even without CWD mortality, we predicted that this population would not grow under current conditions. This finding was unremarkable considering mule deer populations throughout North America are underperforming in the absence of CWD [43]. Chronic wasting disease may exacerbate population declines in herds that are currently considered CWD-free. From 2010–2014, we predicted the southern Converse County herd would decline by >50% using our estimated *λ* = 0.79 and a starting population size of ~6,100 deer. Earlier models of CWD epidemics in mule deer using prevalence observed in our study herd forecasted similar dramatic outcomes [44,45]. This population has experienced population declines of approximately 50% based on WGFD population estimates prior to the start of our study [19]. However, this population did not appear to decline as dramatically during the study as our estimate of *λ* would suggest based on WGFD population estimates (approximately a 4% decline from 2010 to 2014) [19]. While the 2010 and 2014 population size estimates were not strikingly different, the general trend over time suggests a declining population. From 2011 to 2012, WGFD estimated a 19% decline in mule deer numbers and a 15% decline the following year [19]. These declines observed during our study fall within our 95% CI for *λ* (0.72, 0.87). In 2013, greater spring precipitation ended a year-long drought and moderate winter conditions resulted in a 5% increase of the population estimate in 2014 [19]. Therefore, while the population experienced productive years and deer numbers increased; these increases were marginal compared to the larger declines observed over multiple years.

We did not find disease-associated declines in reproduction for mule deer, nor have they been observed in sympatric white-tailed deer [40]. Females were pregnant regardless of CWD status during captures when they were approximately 75 days bred. Despite evidence that suggests CWD-positive mule deer recruit fewer fawns than CWD-negative deer [11], we did not detect a difference in fawn recruitment based on CWD status. Even with a reduction in fawn recruitment of CWD-positive mule deer in Colorado, inclusion of this vital rate in models did not significantly influence *λ* [11]. While CWD did not have a detectable impact on annual pregnancy and recruitment, lifetime reproduction of prime-aged females was likely reduced due to increased annual mortality of CWD-infected individuals. Prion-infected Table Mesa mule deer in Colorado survived an additional 1.6 years on average compared to 5.2 years for uninfected deer [16]. Furthermore, fawns produced by CWD-negative deer, which more likely possessed the more resistant genotype compared to CWD-positive deer in our study, potentially contributed to the increase of the F allele in the population.

Prion protein genotype was important in determining CWD infection and influenced *λ* for *Prnp*-specific segments of the population. As was expected, mule deer that possessed the 225SS *Prnp* genotype were more likely to be CWD-positive compared to 225SF and 225FF deer in our study. We only detected one 225SF CWD-positive deer even though 225*F deer comprised 22% of the study population. Two radio-collared 225FF deer were captured in 2013 and survived to study termination in 2014 with negative tonsil biopsy IHC results. However, evidence suggests current IHC techniques may have lower sensitivity in detecting CWD-positive tissues of 225FF mule deer [46]. Both 225FF deer were estimated to be 3.5 years old during their initial capture, both were pregnant in 2013 and 2014, and during 2013 recruitment surveys, one had a single fawn at heel. The other 225FF deer was not observed during 2013 fawn recruitment surveys. During 2014 captures, ultrasound revealed that one 225FF deer was pregnant with twins and the other was pregnant with a single fetus. Based on a small sample size, free-ranging 225FF mule deer appeared to be as ecologically fit as 225SS deer. The few 225FF mule deer observed in captivity were characterized as atypical in behavior, body condition, and reproductive performance [46]. Formal investigations looking at the effects of *Prnp* genotype on fitness are necessary to determine how populations with greater numbers of 225FF mule deer will persist despite their reduced susceptibility to CWD.

Estimates of *λ* for 225SS and 225*F segments of the population were mediated by varying CWD incidence rates. Using 225SS CWD incidence in our matrix model, we estimated an annual population decline of 33% of 225SS deer. A model incorporating 225*F CWD incidence estimated an annual population decline of 1%. These results suggest the 225*F segment of the population was nearly stable while the 225SS segment of the population was declining rapidly. Using previously published data of mule deer genotyped in the early 2000s from the same geographic area [15], we estimated a 10% population increase in the F allele frequency in less than 10 years [47]. Other factors were not identified that may potentially increase F allele frequency in the absence of CWD as it was outside of the scope of our study. Adaptation to CWD has previously been demonstrated in elk [48] and white-tailed deer [49] using empirical data and statistical models.

Natural selection in favor of less susceptible *Prnp* genotypes may be assisted with selective predation by mountain lions and harvest by hunters of prion-infected deer. While CWD-positive deer were more likely to be killed by mountain lions compared to uninfected deer, it is not clear if this source of mortality regulated or influenced the observed CWD epidemic. Selective predation of CWD-positive deer in Table Mesa, Colorado did not appear to control CWD transmission [16] and it also did not appear to curtail CWD prevalence in the current study herd. Theoretic modeling incorporating 15% predation rate and four times greater risk of predation of prion-infected deer resulted in the eradication of CWD in a closed population [50]. While we observed one year of 15% predation of marked deer in 2010, 3–4% predation rate was typical for most years of the study and it never exceeded 15%. While direct mortality could decrease the subset of infected animals in a population, predators may also act as mechanical vectors that spread prions across the landscape. Infectious prions were demonstrated to pass through the digestive system of coyotes (*Canis latrans*) three days post ingestion suggesting the potential role of carnivores in prion transport and spread [51]. At this time, empirical evidence that supports a predator influence on CWD epidemics does not exist. However, with the expected spread of CWD into areas such as the Greater Yellowstone Area that is occupied by several large predators (i.e. wolves (*Canis lupus*), grizzly bears (*Ursus arctos*), and mountain lions), the role of predators in prion transmission dynamics may soon become more relevant [50]. A multi-predator system may have a greater impact on an emerging CWD epidemic, especially before significant prion contamination occurs in the environment.

Hunting mortality was minimal in our study, although it appeared that sympatric CWD-positive mule deer and white-tailed deer were selectively harvested [13]. It is unclear why others have found no difference in hunting risk between infected and uninfected deer [52], but it is logical that CWD-positive individuals are more vulnerable to harvest due to behavioral changes associated with the disease. The precipitous decline in survival of CWD-positive males increased predictably during the short hunting season around day 250. However, unpredictably there was an observed accelerated decline in the survival curve of CWD-positive females after day 275. Multiple factors may have contributed to greater mortality of CWD-positive females on winter range including increased risk of predation and stressors associated with the rut, hunting season, recruitment of fawns, and winter conditions. Regardless of the cause, CWD-positive deer were more likely to die on winter ranges. This has important implications for the spread and translocation of CWD across the landscape. Congregating deer on winter range may act as a source for CWD-infection to disparate populations when deer migrate in the spring to different summer ranges. These temporal behaviors could explain some of the spatial heterogeneity of CWD prevalence across the landscape [53].

Without an effective CWD vaccine or treatment, management of this disease is limited to focusing on those individuals that are not yet prion-infected. According to our sensitivity analysis, changes in CWD-negative adult survival would cause the greatest changes in *λ*. Improving survival of uninfected mule deer may partially mitigate the impact of CWD. However, to achieve close to stable population growth rates required an unrealistic scenario of 100% survival of CWD-negative deer under high CWD prevalence conditions. We observed low fawn recruitment (0.51 fawns) during the study regardless of disease status compared to an adjacent herd (0.68 fawns) located north of SCMDH [54] and populations throughout the species range (> 0.75 fawns) [11,55]. Management strategies that focus on improving both adult survival of CWD-negative deer and fawn recruitment may increase *λ*. Mule deer populations that currently experience low adult and fawn survival should be closely monitored for CWD because our models predicted less than ideal outcomes once CWD was established.

Lastly, we predicted stable population growth only when CWD prevalence was reduced to 0% in our model. Eradication of CWD is an improbable goal in endemic areas, especially where CWD has been detected for over a decade and potentially present for over 50 years [45]. However, these findings highlight the importance of preventing or slowing the spread of CWD to naïve populations. Mule deer populations currently undergoing declines in the absence of CWD, such as in Nevada and South-central British Columbia [56,57] and in western Wyoming, should be routinely surveyed for detection of CWD. Intensive surveillance that could detect the first few positive CWD cases and rapid removal of prion-infected individuals may be the difference between an established epidemic and local CWD eradication as apparently accomplished in New York and Minnesota [58,59]. While most state agencies focus efforts on collecting hunter harvested and road-killed deer for CWD testing, we recommend incorporating predator-killed deer to the repertoire based on our finding of greater susceptibility of CWD-positive deer to predation [16,17]. Many other non-disease-associated factors contribute to declining mule deer populations and CWD could be the fatal consequence for many herds. Due to the lack of effective management tools to eliminate CWD once established, we suggest management focus efforts and research on how to slow or potentially prevent the movement of CWD across the landscape into uninfected populations.

## Conclusions

With this study, we have demonstrated the long-term consequences of endemic CWD on a free-ranging mule deer population. Chronic wasting disease caused significant declines in the study mule deer herd as well as in sympatric white-tailed deer [13]. Unlike sympatric white-tailed deer, where removal of female harvest may permit *λ* to increase to stable levels based on model estimates [13], elimination of the mule deer doe/fawn hunting season prior to the onset of our study did not result in *λ* ≥ 1. A limited antlered-only harvest in this herd provides a reliable source for monitoring short-term CWD prevalence trends [53]. Additionally, improving and conserving critical mule deer habitats may diminish the negative impacts of CWD, but will not completely mitigate the undesirable population effect of CWD based on our model outcomes. Lastly, without the use of effective vaccines, treatments, and sustainable techniques to reduce CWD incidence, management can currently only focus on slowing the spread of CWD to CWD-free populations.
